# Lumbrokinase, a Fibrinolytic Enzyme, Prevents Intra-Abdominal Adhesion by Inhibiting the Migrative and Adhesive Activities of Fibroblast via Attenuation of the AP-1/ICAM-1 Signaling Pathway

**DOI:** 10.1155/2023/4050730

**Published:** 2023-01-12

**Authors:** Que Thanh Thanh Nguyen, Hyemin Rhee, Mikyung Kim, Moo Yeol Lee, Eun-Ju Lee

**Affiliations:** ^1^Department of Obstetrics and Gynecology, Chung-Ang University School of Medicine, Seoul 06974, Republic of Korea; ^2^Department of Physiology, Chung-Ang University School of Medicine, Seoul, Republic of Korea; ^3^Department of Pathology, Chung-Ang University School of Medicine, Seoul, Republic of Korea

## Abstract

Intra-abdominal adhesion is a complication following abdominal surgery caused by the suppression of fibrinolytic activity and aggravated fibroblast invasion of the injured area, which may lead to chronic illnesses such as chronic pain, intestinal obstruction, and female infertility. This study hypothesized that lumbrokinase, a fibrinolytic enzyme extracted from the earthworm, supports the wound healing process. Therefore, we assessed the effect of lumbrokinase on intra-abdominal adhesion. Lumbrokinase treatment significantly decreased the severity and the area of intra-abdominal adhesion *in vivo* in a dose-dependent manner compared with the controls (untreated and hyaluronate-treated). Lumbrokinase-associated adverse effects were not observed. Immunohistochemical analysis of adhesion tissues revealed a loosened adhesive band between tissues, coupled with significantly decreased peritoneal thickening in the lumbrokinase-treated group versus the control group. Three-dimensional spheroid, MTT, and scratch wound migration assays using the IMR-90 human fibroblast cell line demonstrated that lumbrokinase significantly attenuated the migration and adhesive activity of fibroblasts without compromising cell proliferation. The luciferase assay and western blot analysis showed that lumbrokinase inhibited the AP-1/ICAM-1 cell adhesion signaling pathway. Therefore, lumbrokinase decreases intra-abdominal adhesion and peritoneal thickening by augmenting fibrinolytic action and inhibiting fibroblast migration and adhesive activity via attenuation of the AP-1/ICAM-1 signaling pathway. Lumbrokinase is thus a promising agent to prevent intra-abdominal adhesion.

## 1. Introduction

Postsurgical adhesion is a consequence of tissue injury and the wound healing process. Approximately one-third of patients that underwent laparotomy or laparoscopy were readmitted for complications directly or indirectly related to adhesions [1]. Postsurgical adhesions increase operation time and the risk of bowel injury during subsequent surgery [2, 3] and are a major cause of unsolved chronic problems such as chronic pain, intestinal obstruction, urinary dysfunction, and female infertility [4]. Anti-inflammatory agents or various adhesion barriers have been used to minimize postsurgical adhesion. However, there is insufficient evidence that such approaches improve or prevent postsurgical adhesion [5].

Intra-abdominal adhesions are pathological fibrous connections bridging peritoneal surfaces due to incomplete peritoneal repair. Following peritoneum injury, the fibrin-containing exudates of cells (e.g., leukocytes and macrophages) are deposited in the injured area to prevent bleeding, and a preliminary fibrin scaffold is quickly formed. Most fibrinous exudates are transient and are normally broken down by fibrinolysis within 72 h [6]. Healing then occurs through a combination of fibrosis and mesothelial regeneration. However, trauma-induced local suppression of fibrinolytic activity leads to early fibrinous adhesions, which is soon followed by the invasion of fibroblasts and blood vessels, resulting in permanent adhesions [7]. The proliferating fibroblasts exacerbate matrix invasion, continuously organizing and depositing collagen in the extracellular matrix and establishing mature, fibrous adhesions [8, 9]. Therefore, the recovery of fibrinolytic activity in injured sites and blocking early migration and excessive invasion by fibroblasts are important to prevent postsurgical adhesion.

Lumbrokinase is a 25–32 kDa fibrinolytic enzyme extracted from earthworms [10, 11]. Due to its effective fibrinolytic activity and inhibitory effects on platelet aggregation, lumbrokinase has been clinically proven to prevent and treat cardiovascular disease, and its amyloid degrading potential was recently reported [12–15]. Although lumbrokinase has a similar fibrinolytic effect to streptokinase or urokinase, which mediate the conversion of inactive plasminogen to active plasmin, possibly leading to systemic bleeding [16], lumbrokinase does not activate plasminogen into plasmin [17], thereby reducing the risk of hemorrhage. Moreover, unlike trypsin, which catalyzes the hydrolysis of peptide bonds and breaks down proteins such as fibrin clots [18], lumbrokinase shows high specificity for fibrin [17] and therefore does not induce hyperfibrinolysis. Given that impaired fibrinolytic activity of the injured peritoneum is the main reason for adhesion formation, lumbrokinase might reduce intra-abdominal adhesion by intensifying the fibrinolytic activity of the injured peritoneum. To investigate the potential of lumbrokinase as an antiadhesion agent to prevent postoperative tissue adhesion, we investigated the effect of lumbrokinase on intra-abdominal adhesion by establishing *in vitro* and *in vivo* study models and demonstrated its molecular mechanism.

## 2. Materials and Methods

### 2.1. Preparation of Rats

Male Sprague-Dawley rats weighing 150 to 200 g were purchased from Young Bio (Seoul, Korea). The rats were housed at room temperature (24 ± 2°C) and fed with standard rat chow and provided with drinking water *ad libitum*. All rats were acclimatized for at least seven days before the experiment, and they were allowed to fast 12 h before the procedure. This study was approved by the Institutional Animal Care and Use Committee of Chung-Ang University (Approval No. 2016-00081).

### 2.2. Lumbrokinase and Hyaluronate

Lumbrokinase was purchased from Canada RNA Biochemical Inc. (Richmond BC, Canada), and the hyaluronic acid and sodium carboxymethyl cellulose gel (HA; Guardix-sol^Ⓡ^) was purchased from Hanmi Inc. (Seoul, Korea). The lumbrokinase was diluted with 0.9% NaCl normal saline solution or HA. Before applying the solution to the injury site, the solutions were prepared using identical syringes numbered for identification. To avoid selection bias, each treatment was randomly numbered and the experimenter did not know the content of each bottle. Therefore, the solutions were blindly applied to the rats.

### 2.3. Operation Procedure for Adhesion Formation

After anesthetizing the rats via enflurane inhalation (Gerolan^Ⓡ^ solution, Joongwae Pharmaceutical Co., Seoul, Korea), the entire abdominal skin was disinfected with povidone-iodine under aseptic conditions in a laminar flow hood. The abdominal cavity was accessed through a midline 8 to 10 cm long skin incision along the linea alba. To generate a dense and wider adhesion, a nickel coin-sized area of the peritoneum was scratched using sharp forceps to induce spot bleeding and, at the same time, the cecum wall was injured with a cytobrush until petechial spotting occurred. After inducing these tissue injuries, 500 *μ*L of the solution was applied topically to thoroughly cover the injury area. The peritoneum was closed with a continuous suture using 3-0 polyglactin 910 (Ethicon Inc., Somerville, NJ, USA), and the skin was closed with an auto clip (Jeung-Do Bio & Plant, Seoul, Korea). Sterilization with povidone was applied to the suture sites. On the indicated day, a skin incision was made to monitor the adhesion status and to perform an accurate adhesion grading. The tissues were obtained for histopathological analysis.

### 2.4. Assessment of Adhesion

To determine the optimal time for evaluating tissue adhesion, adhesion formation was observed 1, 2, and 3 weeks after the tissue injuries. One week after the tissue injuries, histological analyses indicated that fibroblasts and inflammatory cells were accumulated in the adhesion tissues during the healing process. In contrast, the tissues were completely healed two and three weeks after the tissue injuries (Figure S1). Therefore, all downstream evaluations were conducted at two weeks postinjury.

An abdominal adhesion was defined as a fibrous attachment between the peritoneum wall and abdominal visceral organs. Attachment of the omentum to the surrounding structures was not considered an adhesion. Two blind observers evaluated the severity and area of the adhesion based on the Linsky scoring system [19], and each score is represented in Figure S2.

### 2.5. Immunohistochemical Analysis

The adherent tissue samples, including the cecum wall and peritoneum, were taken, fixed in 4% formalin, and then, the paraffin-embedded sections were cut to 5 *μ*m in thickness. After dewaxing in xylene and dehydrating in an alcohol gradient, the nuclei were stained with Mayer's hematoxylin solution for 8 min. The slides were then rinsed in tap water until the water was colorless. Next, 1% eosin Y solution was used to stain the extracellular matrix and cytoplasm. After rinsing with tap water, the slides were dehydrated and cleared with an alcohol gradient and xylene, respectively. A coverslip was placed over the section and held in place with a mounting medium. Histological analyses were conducted under light microscopy. For Masson's trichrome staining, the slides were deparaffinized and dehydrated in an alcohol gradient. Then, the slides were refixed in Bouin's fluid at 56–60°C for 1 h and stained in Weigert's iron hematoxylin solution for 10 min after rinsing the slides under running tap water for 5 min to remove the yellow color. Next, the slides were rinsed again and stained in Biebrich scarlet-acid fuchsin solution for 10–15 min. The slides were then transferred directly to aniline blue solution, stained for 5–10 min, briefly rinsed, and differentiated in a 1% phosphomolybdic-phosphotungstic acid solution with 1% acetic acid for 2–5 min. Afterward, the slides were quickly dehydrated through 95% alcohol, wiped with absolute ethyl alcohol to remove the Biebrich scarlet-acid fuchsin staining, and cleared with xylene. A mounting medium was used to bond the coverslip to the slide. Finally, a single-blinded pathologist assessed the histological finding of each slide.

### 2.6. Cell Line

The IMR-90 human fibroblast cell line was purchased from the Korean Cell Bank (Seoul, Korea). The cells were maintained in minimum essential medium (Welgene, Gyeongsan, Korea) with 10% fetal bovine serum (FBS), 100 U/mL penicillin, and 100 *μ*g/mL streptomycin (Invitrogen, Carlsbad, CA, USA). All cells were kept at 37°C in a humidified atmosphere with 5% CO_2_. The cell media was changed every three days.

### 2.7. MTT Assay

The 3-(4,5-dimethylthiazol-2-yl)-2,5-diphenyltetrazolium bromide (MTT) assay was performed to assess cell viability in both two-dimensional (2D) and three-dimensional (3D) cultures. The MTT reagent (12 mM) was added to the cells and incubated for 3 h according to the manufacturer's instructions (M6494, Invitrogen, Eugene, OR, USA). Afterward, 100 *μ*L of dimethyl sulfoxide was added to dissolve the generated formazan crystals (i.e., the MTT reaction product) at room temperature for 30 min. Cell viability was then estimated by measuring the sample absorbance at 540 nm using an enzyme-linked immunosorbent assay (ELISA) reader (Epoch2, BioTek; Winooski, VT, USA). The experiment was performed in triplicate and repeated at least three times.

### 2.8. 3D Spheroid Assay and Cell Adhesion Assay

IMR-90 cells were cultured in a microwell array, as described previously [20]. Briefly, the cells were loaded into a microwell array at a 5 × 10^5^ cell/mL density, and approximately 3 × 10^3^ cells were present in each microwell. The cells aggregated after 20 min of seeding, which was indicative of spheroid formation. Then, the IMR-90-forming spheroids were incubated with the media with or without lumbrokinase (2,000 U/mL), and half of the medium was replaced every day. After 14 days, the spheroids in each well were imaged using light microscopy (4x magnification; scale bar, 300 *μ*m) to compare the spheroid formation ability of the two groups. To assess the viability of the cell spheroids, the MTT assay was performed after preparing a single-cell suspension. To assess the cell adhesion ability, the spheroids were transferred to a 2D surface-coated culture plate. After incubation for 24 h, the media was changed, and the attached cells were counted after 14 days.

### 2.9. Scratch Wound Migration Assay

Cell migration was determined by a scratch wound migration assay. IMR-90 cells were seeded onto 6-well plates at 80%–90% confluence. After 24 h of seeding, the cells were washed with phosphate-buffered saline, scratched with a 1,000 *μ*L pipette tip, and incubated in media with or without lumbrokinase at 1,000 and 2,000 U/mL. Cell motility was tracked 24 h posttreatment, and cells migrating from the basal line were observed. The migrating cells were calculated as the number of cells in the recovered area compared to the initial gap at 0 h. Images were captured using a light microscope (40x magnification; scale bar, 200 *μ*m). The migrated cells were then counted using the ImageJ software (NIH Image Processing and Analysis in Java).

### 2.10. Dual-Luciferase Reporter Assay

The dual-luciferase assay was performed using the Dual-Luciferase Reporter Assay System kit (Promega; Madison, WI). Each experiment was performed in triplicate. Briefly, the cells were cotransfected with a 10 : 1 ratio of transcription factors produced by firefly luciferase and *Renilla* luciferase (pRL-SV40). The cells were harvested and dissolved in 200 *μ*L of passive lysis buffer. Lysates were cleared by centrifugation at 14,000 rpm for 15 min, and 20 *μ*L of each cell extract was transferred to a 1.5 mL centrifuge tube containing 100 *μ*L/tube of the provided Luciferase Assay Reagent II. The provided Stop and Glo Reagent (100 *μ*L/tube) was then added to initiate *Renilla* luciferase activity, and the ratio of firefly luciferase activity to *Renilla* luciferase activity was calculated.

### 2.11. Western Blot Analysis

The western blot analyses were performed as previously described [21]. The following antibodies were used for the western blot analysis: anti-c-Jun (sc-166540), anti-phospho-c-Jun (sc-822), anti-intracellular adhesion molecule-1 (anti-ICAM-1) (sc-8439), and anti-*β*-actin (all from Santa Cruz Biotechnology, Santa Cruz, CA, USA). *β*-Actin was used as the loading control. Phorbol 12-myristate 13-acetate (PMA; Glentham Life Science, Corsham, UK) and T-5224 (MedChemExpress, NJ 08852, USA) were used to enhance and inhibit AP-1 activation, respectively. The protein bands in the western blot films were quantified with the ImageJ software (NIH). All experiments were performed in triplicate and repeated at least three times.

### 2.12. Statistical Analysis

The data of the two groups were compared using Student's *t*-test or the Mann-Whitney test for continuous variables. The Kruskal-Wallis *H* test was used to compare the adhesion scores among the experimental groups. All reported *p* values are two-sided; a *p* value less than 0.05 was considered statistically significant. All analyses were conducted using the SPSS software version 17.0 (SPAA Inc., Chicago, IL, USA).

## 3. Results

### 3.1. Lumbrokinase Reduces Intra-Abdominal Adhesion

To assess the optimal time to evaluate intra-abdominal adhesion in rats, we evaluated the adhesion status one, two, and three weeks after inducing the tissue injuries (Figure S1). The gross evaluation showed that serious adhesions were well established in all rats, but no perforation was found, indicating an adequate and safe degree of tissue injuries. Histologic evaluation revealed the accumulation of inflammatory cells and fibroblasts at the adhesion area one week after tissue injury, suggesting that the healing process was in the inflammatory and proliferation phase. The adhesion area two and three weeks after the tissue injuries exhibited the characteristic hallmarks of the late stage of wound healing with collagen synthesis. Therefore, we concluded that the optimal time to observe adhesion was two weeks after tissue injury.

From the result of the fibrin lysis assay (Figure S3), we determined that 5,000 U/mL was the minimal effective concentration of lumbrokinase. Therefore, lumbrokinase concentrations of 5,000, 10,000, and 15,000 U/mL were evaluated. HA has been widely used in the clinical field to prevent postoperative adhesion, and therefore, the nontreated and HA-treated groups were used as controls. Each group had four subjects, and the adhesion status was assessed two weeks after lumbrokinase treatment. The adhesion score in the control was grade three of severity, covering >50% of the injured tissue area in all rats, thus confirming the successful production of adhesion formation (Tables 1 and 2). The severity and area of adhesions were significantly reduced in the lumbrokinase-treated group compared with the control group (*p* = 0.040 and *p* = 0.009, respectively). The adhesion score was significantly lower in the HA+lumbrokinase-treated group than in the group treated with HA without lumbrokinase (*p* = 0.014 and *p* = 0.007, respectively).

### 3.2. Lumbrokinase Causes Loosening of Adhesion Band

**Figure 1:**
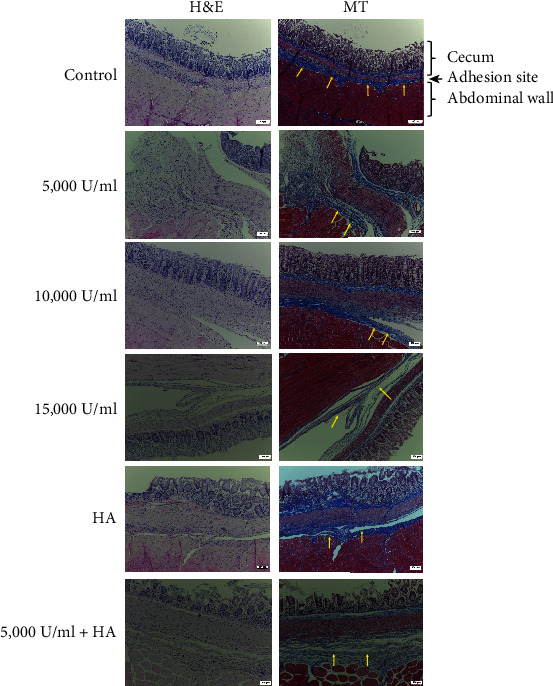
Loosening of adhesion in the central area. Hematoxylin and eosin (H&E) and Masson's trichrome (MT) (40x) staining were performed in the tissue samples obtained after two weeks of lumbrokinase application. Masson's trichrome stain showed fibrosis (blue-stained), which represented the adhesion site between the cecum and abdominal wall. The arrows indicate fibrosis of the peritoneal wall side. Scale bar, 100 *μ*m.

To assess the effect of lumbrokinase on adhesion through microscopy observations, the adherent tissue samples containing the cecum wall and peritoneum were collected. Rats with a severe score for adhesion in each group were selected. Immunohistochemical analysis showed that the adhesion was tight in the entire adhesion lesion of control rats. In lumbrokinase-treated rats, the edges of the adhesion lesion between the cecum and the abdominal wall were tightly adherent, similar to the control rats. However, the central area was loosened and detached (Figure 1). A severe score was assigned because of the dense attachment of the edge of the adhesion area. The loosening of the central area was likely caused by the decreased fibrin deposition, providing evidence of the inhibitory effect of lumbrokinase.

### 3.3. Lumbrokinase Reduces Peritoneal Thickness

**Figure 2:**
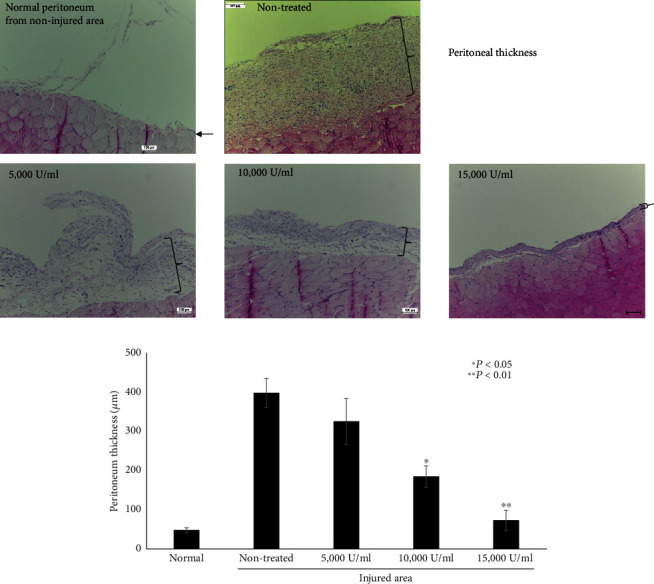
Peritoneal thickness in normal and injured areas. (a) After one week of injury, peritoneum tissue from noninjured and injured areas was obtained and stained with hematoxylin and eosin (H&E). The reduction in peritoneal thickness occurred in a dose-dependent manner. Scale bar, 100 *μ*m. A monolayer of mesothelial cells was observed in the normal peritoneum from the noninjured peritoneum (arrow). The peritoneum of the injured area was thickened in the nontreated group, whereas peritoneal thickness was significantly reduced in the lumbrokinase-treated groups. (b) Quantification of the peritoneal thickness. The thickness measurement is the average obtained at four different sites in each rat based on image analysis with the ImageJ software (*p* value for the indicated groups compared with the control).

Peritoneal inflammation leads to the exfoliation of mesothelial cells and the thickening of the submesothelium, thus affecting peritoneal thickness. To assess the effects of lumbrokinase on the injured peritoneum, we assessed the peritoneal thickness after peritoneal injury in two rats in each group (Figure 2). One week after tissue injury, peritoneal tissues were collected from the injured site. Normal peritoneum tissues without tissue injuries, which were obtained from the nontreated rats, were covered with a monolayer of mesothelial cells. The injured peritoneum of the nontreated rat was thickened and enriched with the infiltration of numerous cells. In contrast, the thickness of the injured peritoneum in the lumbrokinase-treated rat was significantly decreased in a dose-dependent manner.

### 3.4. Lumbrokinase Has No Negative Hematological Effects

Enhanced fibrinolytic activity is linked to postsurgery bleeding. Therefore, we assessed the coagulation and hematological profiles on day 7 after the intraperitoneal application of lumbrokinase. The evaluation was performed on 14 rats, which were divided into two groups. To assess the coagulation profile, the bleeding time and prothrombin time were measured in seven rats and our findings indicated that these endpoints were not significantly affected by lumbrokinase and HA treatment (Figure S4). Additionally, there were no differences in the red blood cell count, hemoglobin, and platelet count. The rats treated with high doses (10,000 and 20,000 U/mL) of lumbrokinase exhibited leukocytosis due to lymphocytosis (Table S1). However, these were within normal ranges [22]. Feeding difficulty, death, cecum perforation, severe peritonitis, and other complications were not found. Moreover, none of the rats exhibited any adverse effects.

### 3.5. Lumbrokinase Inhibits the Migration and Adhesion of Fibroblast Cells with No Effect on Proliferation

**Figure 3:**
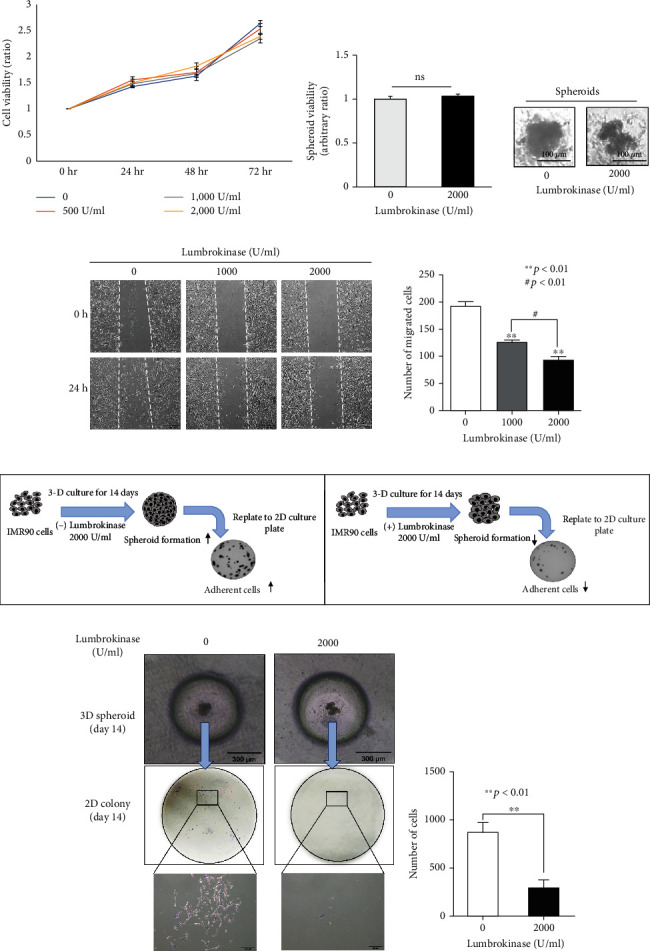
Lumbrokinase inhibits the migration and adhesive ability of fibroblast cells. (a) IMR-90 cells were seeded at a density of 1 × 10^4^ cells/well of a 96-well plate. One day after seeding, the cells were treated with the indicated concentrations of lumbrokinase. After treatment for 24, 48, and 72 h, cell viability was measured by the MTT assay and cell viability ratios were calculated as the ratio of the OD540 value of the lumbrokinase treatment groups relative to the OD540 value of the untreated control (0 U/mL) following each time point. The experiment was performed in triplicate and repeated at least three times. (b) IMR-90 cells (5 × 10^5^ cells/mL) were loaded in the microwell array. The cells aggregated after 20 min of seeding, which was indicative of spheroid formation. One day after seeding, the cells were treated with lumbrokinase every three days. The viability of cell spheroids was measured 14 days later using the MTT assay after preparing a single-cell suspension (left panel, ns, not significant). The shape of the spheroids from lumbrokinase-treated cells was distorted, whereas the control spheroids were circular in shape (right panel). (c) The effect of lumbrokinase on fibroblast cell motility was evaluated by the scratch wound migration assay. After lumbrokinase treatment at the indicated concentration for 24 h, migrated cells were counted based on images generated with the ImageJ software (^∗∗^*p* < 0.01 vs. control (0 U/ml); ^#^*p* < 0.01 indicates a significant difference between the 1,000 and 2,000 U/mL lumbrokinase-treated groups). (d) Schematic diagram of the experimental protocol to assess fibroblast adhesive ability under lumbrokinase treatment. (e) Three-dimensional spheroids were made for 14 days after lumbrokinase treatment. Photos were taken under light microscopy. The spheroids were separated into single cells, after which these cells were recultured in a two-dimensional 6-well culture plate. The number of attached cells was counted under a light microscope on day 14.

Mesothelial cells are lost upon peritoneal injury, and peritoneal fibroblasts play key roles in inflammation and immune cell recruitment to sites of tissue injury and as a source of inflammatory mediators [23]. The fact that lumbrokinase reduced peritoneal thickness prompted us to assess the effect of lumbrokinase on fibroblasts. We first assessed cell viability after lumbrokinase treatment; lumbrokinase did not influence fibroblast cell proliferation in both 2D (Figure 3(a)) and 3D spheroid cultures (Figure 3(b)). Notably, spheroids produced from lumbrokinase-treated cells were distorted in shape in contrast to the circular control spheroids (Figure 3(b)). The cell migration assays showed that the number of migrated cells in the lumbrokinase-treated group was significantly low compared with those in the nontreated group, and the reduction of migrated cells occurred in a dose-dependent manner (Figure 3(c)). Despite the different features of the spheroids, the cell viability of the spheroids in the two groups was not significantly different. This finding suggests that the cells treated with lumbrokinase might lose cell-cell adhesion ability. To further prove whether lumbrokinase compromises cell-to-cell adhesion, we replated the 3D spheroids onto a commercial coated 2D culture plate as illustrated in Figure 3(d). Our findings demonstrated that the number of lumbrokinase-treated cells attached to the surface of the cell culture plate was significantly lower compared with that of the nontreated cells (Figures 3(e) and 3(f)).

### 3.6. Lumbrokinase Inhibits the AP-1/ICAM-1 Signaling Pathway

**Figure 4:**
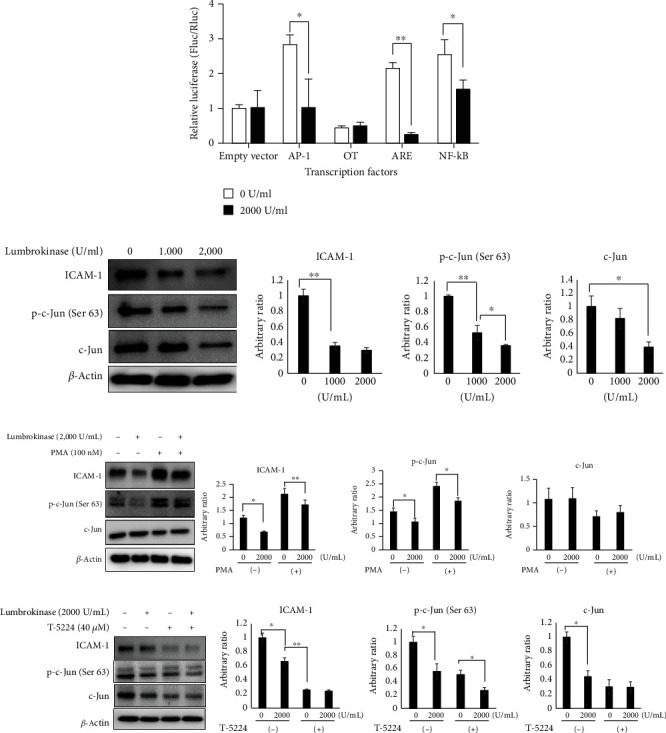
Lumbrokinase inhibits the AP-1/ICAM-1 signaling pathway. (a) IMR-90 cells (1 × 10^5^) were seeded into a 24-well culture plate. One day after incubation, the cells were treated with lumbrokinase (2,000 U/mL) for two days. The cells were then transfected with reporter plasmids containing the promoter of AP-1, *β*-catenin (OT), ERK (ARE), and NF-*κ*B. The cells were harvested two days after transfection, and the transcriptional activity was measured using a dual-luciferase reporter assay kit. ^∗∗^*p* < 0.01 and ^∗^*p* < 0.05. (b) 5 × 10^5^ IMR-90 cells were seeded in 6-well plates, then treated with the indicated concentration of lumbrokinase after 24 h. After 48 h of incubation, the cells were harvested for western blot analysis. (c) 5 × 10^5^ IMR-90 cells were incubated with or without lumbrokinase (2,000 U/ml) in the presence and absence of phorbol 12-myristate 13-acetate (PMA), an AP-1 enhancer, for 48 h. Then, the cells were harvested and subjected to western blotting. The graph was plotted based on the band densities measured using the ImageJ software. ^∗∗^*p* < 0.01 and ^∗^*p* < 0.05. (d) 5 × 10^5^ IMR-90 cells were plated and treated with lumbrokinase (2,000 U/mL) and T-5224 (an AP-1 inhibitor) 24 h later. The cells were harvested after 48 h of incubation in their respective, after which western blotting was performed. The graph was plotted based on the band densities measured using the ImageJ software. ^∗∗^ *p* < 0.01 and ^∗^*p* < 0.05.

To understand the underlying mechanism of the antiadhesive effect of lumbrokinase, we next screened transcription factors involved in cell adhesion, including activator protein 1 (AP-1), *β*-catenin, extracellular signal-regulated kinase (ERK), and nuclear factor kappa B (NF-*κ*B) [24–27]. Our findings demonstrated that the transcriptional activities of ERK, AP-1, and NF-*κ*B were significantly reduced in fibroblast cells with lumbrokinase treatment compared to those without treatment, whereas the transcriptional activity of *β*-catenin was not different between the two groups (Figure 4(a)). AP-1 is a downstream target of ERK [27], and therefore, our observations promoted us to further assess the regulatory association between AP-1 and lumbrokinase. ICAM-1, a protein downstream of AP-1 [28], is associated with the collective invasion of fibroblast by mediating the actomyosin contractility of fibroblast [29] and may serve as a marker to identify proinvasive stromal fibroblasts. Therefore, we assessed whether lumbrokinase reduces the AP-1 protein and its downstream protein, ICAM-1. Western blotting analyses demonstrated that lumbrokinase downregulated AP-1 and phosphorylated AP-1 and also reduced ICAM-1 in a dose-dependent manner (Figure 4(b)). The c-Fos protein was not detected in IMR-90 fibroblast cells because c-Jun is the predominant AP-1 in fibroblast. Quiescent fibroblasts have mainly c-Jun homodimers, and exponentially growing fibroblasts exhibit Fra : c-Jun heterodimers [30].

The AP-1 signaling pathway was stimulated using PMA, an AP-1 enhancer, to further assess the effect of lumbrokinase on AP-1 and ICAM-1 in fibroblast cells. Western blot analyses demonstrated that PMA induced upregulation of phospho-c-Jun and ICAM-1 and that upregulation of both proteins was attenuated by lumbrokinase treatment (Figure 4(c)). We then blocked AP-1 by using T-5224, a specific AP-1 inhibitor [31]. T-5224 inhibited c-Jun, resulting in a marked reduction in ICAM-1 expression (Figure 4(d)). This finding indicated that ICAM-1 is dominantly regulated by AP-1. Additionally, ICAM-1 was not further inhibited by lumbrokinase. Taken together, our finding demonstrated that lumbrokinase downregulates the ICAM-1 via the AP-1 signaling pathway. Considering that ICAM-1 plays important roles in cell adhesion and migration [32], lumbrokinase inhibits the adhesion and migration of fibroblast cells by negatively targeting AP-1/ICAM-1.

## 4. Discussion

Although lumbrokinase is a known fibrinolytic enzyme, it has not yet been studied in the context of tissue adhesion. The present study demonstrated that lumbrokinase has a significantly better preventive effect on intra-abdominal adhesion compared with hyaluronate treatment as well as no treatment. Furthermore, our *in vitro* and *in vivo* data showed that the inhibitory effect of lumbrokinase on postoperative adhesion involved reducing the diminished fibrinolytic activity and attenuating the migration and adhesive activity of fibroblasts. At the intracellular level, our findings demonstrated that lumbrokinase modulates fibroblast activity by inhibiting AP-1, a transcriptional factor of ICAM-1, which in turn downregulated ICAM-1, an adhesion molecule. Therefore, lumbrokinase is a promising therapeutic agent to prevent intra-abdominal adhesion after abdominal surgery.

Postoperative intra-abdominal adhesion is caused by an abnormal healing process in which deficient fibrinolytic activity in the injured area leads to early fibrinous adhesion [7]. Accordingly, an increase in fibrinolytic activity was demonstrated to reduce adhesion formation [33]. In our study, in contrast to the dense attachment of the entire adhesion area in the control rats, the loosening of adhesion in the central area in the lumbrokinase-treated group indicated that adhesion formation was compromised. This could be due to the effect of lumbrokinase, which immediately covered the injured peritoneum and the surface of the cecum and rescued the decreased fibrinolytic activity in the early stages of wound healing.

This study demonstrated that lumbrokinase has an appreciable effect on peritoneal wound healing. Peritoneal thickness was significantly decreased in a dose-dependent manner in the rats treated with lumbrokinase compared with the control rats. Throughout the wound healing process, an inflammatory and fibrinous exudate was deposited at the site of peritoneal trauma. If fibrinolytic activity is deficient, fibroblasts invade the fibrin-/fibronectin-rich provisional extracellular matrix, leading to collagen deposition. Finally, structural alterations such as thickening are generated [33, 34]. Therefore, the reduced peritoneal thickness could be due to the early recovery of fibrinolytic activity by lumbrokinase, leading to the inhibition of fibroblast migration and eventually suppressing collagen deposition. This finding demonstrated the beneficial effect of lumbrokinase on wound healing in the peritoneum.

Fibroblast migration plays an important role in dense adhesion, and peritoneal thickening involves fibroblast recruitment [34, 35]. Lumbrokinase induced loosening of the adhesion site and decreased peritoneal thickening, suggesting that lumbrokinase affects fibroblast migration and recruitment. Our study demonstrated that lumbrokinase diminishes the adhesion and motility of fibroblast cells without compromising cell proliferation. Given that excessive fibroblast invasion in the initial step of the wound healing process is critical for adhesion formation, the modulation of fibroblasts by lumbrokinase might be a crucial mechanism for preventing adhesion. Therefore, fibroblast modulation could be a unique function of lumbrokinase, distinguishing it from other fibrinolytic enzymes.

In contrast to our study, a previous report demonstrated that earthworm extract applied after two days of injury promoted skin wound healing and recruited more fibroblasts in the earthworm group compared with the control group [36]. However, the earthworm extract used in the aforementioned study was an ultrafiltered solution collected from homogenized earthworms, which might contain various amino acids, fatty acids, microelements, lumbritin, lumbrofebrin, terrestrolum brolysin, purine, choline, cholesterin, and vitamins [37]. This suggests that the observed effects could have resulted from a complex synergistic response to various ingredients rather than a single component. In contrast, we studied the effects of pure lumbrokinase on intra-abdominal adhesion, and the lumbrokinase was immediately applied. These discrepancies between the two studies could explain their opposing results.

Several studies have explored the effects of earthworm extract components on cell motility. Similar to lumbrokinase, earthworm (*Eisenia fetida*) fibrinolytic enzyme reduced the migration of MCF-7 breast cancer cells by inhibiting adhesion factor CD44v6 and the focal adhesion kinase (FAK) signaling pathway. However, unlike lumbrokinase, *E. fetida* fibrinolytic enzyme induced cell apoptosis [38]. Conversely, an *in vitro* study demonstrated that the ethanolic extracts of lyophilized earthworm powder induced the migration of RSC96 Schwann cells via activation of plasminogen activators and matrix metalloproteinase- (MMP-) 2 and MMP-9 [39]. Moreover, an *in vivo* study showed that lumbrokinase increased osteoblast activity and migration and reduced osteoblast activity in bone remodeling [40]. Therefore, the function of earthworm extracts might depend on the extracted components, cell types, and pathological conditions, requiring clarification through further studies.

We further demonstrated that the underlying mechanism of the antiadhesive effect of lumbrokinase on fibroblasts was the downregulation of the AP-1/ICAM-1 signaling pathway. ICAM-1 is expressed at basal levels in several cell types, including fibroblasts and leukocytes. Its expression increases during inflammation to facilitate extravasation and migration into the injured area, thereby influencing the wound healing cascade [41]. For example, endothelial ICAM-1 is involved in monocyte recruitment into the wounded tissue, macrophage ICM-1 contributes to wound debridement and resolution of inflammation, and epithelial cell ICM-1 facilitates wound reepithelialization [42]. Therefore, fibroblast ICAM-1 could play a role in fibroblast deposition. By reducing the ICAM-1 expression of fibroblasts, lumbrokinase reduces fibroblast motility and adherent ability and consequently obstructs its recruitment to the injured peritoneum, thus preventing adhesion formation.

Clinical trials have been conducted to assess whether fibrinolytic agents such as streptokinase and urokinase can prevent postsurgical adhesions. Intraperitoneal streptokinase administration after laparotomy significantly decreased the occurrence of postoperative adhesion in a clinical trial [43] as well as in animal studies, whereas urokinase was ineffective on postsurgical adhesion in an animal study [44]. Based on our results, we evaluated lumbrokinase as a potential candidate for adhesion inhibition. The difference between lumbrokinase and streptokinase is that lumbrokinase does not activate plasminogen into plasmin, thus minimizing the risk of systemic bleeding [17]. Therefore, lumbrokinase is a promising antiadhesive material with a low risk of adverse effects. However, similar to streptokinase and urokinase, lumbrokinase raises antigenicity and immunogenicity concerns, which should be solved prior to its clinical application.

In summary, lumbrokinase significantly prevents intra-abdominal adhesion after abdominal surgery by intensifying fibrinolytic activity and inhibiting fibroblast migration. The novel molecular mechanism of lumbrokinase is the downregulation of adhesion protein ICAM-1 via the AP-1 pathway in fibroblasts. Therefore, lumbrokinase could be a promising antiadhesive material to prevent intra-abdominal adhesion after abdominal surgery.

## Figures and Tables

**Table 1:** Severity of adhesion.

Groups	Severity				*p* value
	0	1	2	3	
Control	0	0	0	4	0.040
5,000 U/mL	2	0	0	2	
10,000 U/mL	4	0	0	0	
15,000 U/ml	3	0	0	1	

HA	0	2	0	2	0.014
5,000 U/ml+HA	3	0	0	1	
10,000 U/ml+HA	4	0	0	0	
15,000 U/ml+HA	4	0	0	0	

HA: hyaluronic acid and sodium carboxymethyl cellulose.

**Table 2:** Area of adhesion.

Groups	Area				*p* value
	0%	<25%	25–50%	>50%	
Control	0	0	0	4	0.009
5,000 U/ml	2	0	2	0	
10,000 U/ml	4	0	0	0	
15,000 U/ml	3	1	0	0	

HA	0	2	1	1	0.007
5,000 U/ml+HA	3	1	0	0	
10,000 U/ml+HA	4	0	0	0	
15,000 U/ml+HA	4	0	0	0	

HA: hyaluronic acid and sodium carboxymethyl cellulose.

## Data Availability

The data used to support the findings of this study are included within the article and the supplementary information files.

## Supplementary Materials

Supplementary Materials Supplementary Material and Methods. *Fibrin Plate Assay*. Fibrinogen (#F3879) and thrombin (#T6884-250UN) were purchased from Sigma-Aldrich (St. Louis, MO, USA). Fibrinogen was diluted in phosphate-buffered saline (Welgene, Gyeongsan, Korea) to make 0.3% concentrated fibrinogen, which was incubated at 30°C for 60 min. After completely dissolving the fibrinogen, the fibrinogen solution was filtered through a 0.2 *μ*m filter, and 10 *μ*L of 20 U/mL thrombin was added to 15 mL of the fibrinogen solution. Next, 13 mL of 1% agarose solution was added to 15 mL of the fibrinogen-thrombin mixture [1, 2]. Afterward, 10 mL of the solution was clotted in a 100*π* Petri dish. The plates were allowed to stand at room temperature (25°C) for at least 30 min before being used. After clotting, holes were made using a Pasteur pipette. Next, 50 *μ*L of the indicated concentration of lumbrokinase was dispensed into the holes of the plates, which were then incubated for the indicated hours at 37°C. Plasmin (P1867-500UG, Sigma, St. Louis, MO, USA) was used (0.1 U/mL) as a positive control. The fibrinolytic activity was calculated as the lytic area surrounding each well of the fibrin plate. The diameter of the area was measured with a ruler. *Bleeding Time and Hematological Test.* Bleeding time was measured using primary saline [3]. This test is performed by immersing the tip of the ear in 0.9% saline at 37°C for 2 min. Then, the tip is transfixed with a scalpel blade (approximately 0.5 cm), after which the cut ear tip is resubmerged in the warm saline, and the time to cessation of bleeding is recorded. A normal value is defined as <5 min, and an abnormal value is defined as >15 min. After measuring the bleeding time, whole blood was collected from the carotid artery. The tests of complete blood count and prothrombin time were performed in the laboratory of Chung-Ang University Hospital. Blood was collected into tubes containing ethylenediaminetetraacetic acid (EDTA) and tubes containing sodium citrate. The normal ranges of hematological parameters of male Sprague-Dawley rats are reported in literature [4]. Figure S1: histological analysis of adhesion area between cecum and peritoneum. Two subjects were used in each group. The adhesion areas at one, two, and three weeks after tissue injuries were shown with hematoxylin and eosin (HE) staining (left panels) and Masson's trichrome staining (right panels). Fibroblasts and inflammatory cells (arrow) were accumulated in the adhesion area in the 1-week tissues, and these were resolved in the two- and three-week tissues. This finding indicated that two weeks is the optimal time to observe tissue adhesion. Figure S2: gross scoring of adhesion two weeks after tissue injuries; the degree of intra-abdominal adhesion was measured by scoring the severity and area of adhesion. The severity of adhesion was as follows: 0, no adhesions; 1, filmy adhesions that are easily separated with blunt dissection or traction; 2, dense adhesions that are separated by blunt dissection but partly sharp dissection; and 3, dense vascularized adhesions that are separated by sharp dissection only. The adhesion area of the initially injured area was assessed as follows: 0, no adhesion; 1, <25% of the traumatized area; 2, 25–50% of the traumatized area; and 3, >50% of the traumatized area. Figure S3: fibrinolytic activity of lumbrokinase. (A) To validate the effect of lumbrokinase, the represented concentration of lumbrokinase was applied to the holes of the fibrin plate and incubated at 37°C for the indicated time. The circles around the hole of the fibrin plate indicated fibrinolysis, and the lysis effect was assessed by measuring the diameter of the circles. Plasmin was used as a positive control. The result showed that lumbrokinase lyses fibrin in a dose-dependent manner. (B) To obtain the minimum concentration to lyse fibrin, low concentrations of lumbrokinase were applied. Treatment with 2,000 U/mL of lumbrokinase had no effect and 3,750 U/mL had minor effects. Therefore, 5,000 U/mL was used as the minimum concentration. Hyaluronate (HA) had no effect on fibrin lysis. (C) To assess whether hyaluronate influenced the lumbrokinase effect, cotreatment of lumbrokinase and hyaluronate was performed. The results showed that HA had no effect and did not compromise the lumbrokinase effect. Figure S4: coagulation profiles after lumbrokinase treatment. Bleeding time (A) and prothrombin time (B) (HA: hyaluronate). Table S1: laboratory results on day 7.

## References

[1] Krielen P., Stommel M. W. J., Pargmae P. (2020). Adhesion-related readmissions after open and laparoscopic surgery: a retrospective cohort study (SCAR update). *The Lancet*.

[2] Coleman M. G., McLain A. D., Moran B. J. (2000). Impact of previous surgery on time taken for incision and division of adhesions during laparotomy. *Diseases of the Colon and Rectum*.

[3] Van Der Krabben A. A., Dijkstra F. R., Nieuwenhuijzen M., Reijnen M. M., Schaapveld M., Van Goor H. (2000). Morbidity and mortality of inadvertent enterotomy during adhesiotomy. *Journal of British Surgery*.

[4] Ten Broek R. P., Issa Y., van Santbrink E. J. (2013). Burden of adhesions in abdominal and pelvic surgery: systematic review and met-analysis. *BMJ*.

[5] Penzias A., Bendikson K., Falcone T. (2019). Postoperative adhesions in gynecologic surgery: a committee opinion. *Fertility and Sterility*.

[6] Beyene R. T., Kavalukas S. L., Barbul A. (2015). Intra-abdominal adhesions: anatomy, physiology, pathophysiology, and treatment. *Current Problems in Surgery*.

[7] Hellebrekers B. W., Kooistra T. (2011). Pathogenesis of postoperative adhesion formation. *The British Journal of Surgery*.

[8] Coccolini F., Ansaloni L., Manfredi R. (2013). Peritoneal adhesion index (PAI): proposal of a score for the "ignored iceberg" of medicine and surgery. *World Journal of Emergency Surgery : WJES*.

[9] Fatehi Hassanabad A., Zarzycki A. N., Jeon K., Deniset J. F., Fedak P. W. M. (2021). Post-operative adhesions: a comprehensive review of mechanisms. *Biomedicine*.

[10] Cho I. H., Choi E. S., Lim H. G., Lee H. H. (2004). Purification and characterization of six fibrinolytic serine-proteases from earthworm *Lumbricus rubellus*. *Journal of Biochemistry and Molecular Biology*.

[11] Fu T., Yang F., Zhu H., Zhu H., Guo L. (2016). Rapid extraction and purification of lumbrokinase from Lumbricus rubellus using a hollow fiber membrane and size exclusion chromatography. *Biotechnology Letters*.

[12] Jin L., Jin H., Zhang G., Xu G. (2000). Changes in coagulation and tissue plasminogen activator after the treatment of cerebral infarction with lumbrokinase. *Clinical Hemorheology and Microcirculation*.

[13] Kasim M., Kiat A. A., Rohman M. S., Hanifah Y., Kiat H. (2009). Improved myocardial perfusion in stable angina pectoris by oral lumbrokinase: a pilot study. *Journal of Alternative and Complementary Medicine*.

[14] Cao Y. J., Zhang X., Wang W. H. (2013). Oral fibrinogen-depleting agent lumbrokinase for secondary ischemic stroke prevention: results from a multicenter, randomized, parallel-group and controlled clinical trial. *Chinese Medical Journal*.

[15] Metkar S. K., Girigoswami A., Bondage D. D., Shinde U. G., Girigoswami K. (2022). The potential of lumbrokinase and serratiopeptidase for the degradation of A*β* 1-42 peptide - an in vitro and in silico approach. *The International Journal of Neuroscience*.

[16] Hollander I. J., Gaffney P. J. (1987). Plasminogen activators and their potential in therapy. *Critical Reviews in Biotechnology*.

[17] Park Y.-D., Kim J.-W., Min B.-G., Seo J.-W., Jeong J.-M. (1998). Rapid purification and biochemical characteristics of lumbrokinase III from earthworm for use as a fibrinolytic agent. *Biotechnology Letters*.

[18] Sarkar N. K. (1960). Mechanism of clot lysis. *Nature*.

[19] Linsky C. B., Diamond M. P., Cunningham T., Constantine B., DeCherney A., diZerega G. (1987). Adhesion reduction in the rabbit uterine horn model using an absorbable barrier, TC-7. *The Journal of Reproductive Medicine*.

[20] Lee S., Kim S., Ahn J., Park J., Ryu B.-Y., Park J. Y. (2020). Membrane-bottomed microwell array added to Transwell insert to facilitate non-contact co-culture of spermatogonial stem cell and STO feeder cell. *Biofabrication*.

[21] Nguyen Q. T. T., Park H. S., Lee T. J. (2022). DKK3, downregulated in invasive epithelial ovarian cancer, is associated with chemoresistance and enhanced paclitaxel susceptibility via inhibition of the *β*-Catenin-P-Glycoprotein signaling pathway. *Cancers*.

[22] He Q., Su G., Liu K. (2017). Sex-specific reference intervals of hematologic and biochemical analytes in Sprague-Dawley rats using the nonparametric rank percentile method. *PLoS One*.

[23] Witowski J., Tayama H., Ksiazek K., Wanic-Kossowska M., Bender T. O., Jörres A. (2009). Human peritoneal fibroblasts are a potent source of neutrophil-targeting cytokines: a key role of IL-1 *β* stimulation. *Laboratory Investigation*.

[24] Weekes D., Kashima T. G., Zandueta C. (2016). Regulation of osteosarcoma cell lung metastasis by the c-Fos/AP-1 target FGFR1. *Oncogene*.

[25] Brembeck F. H., Rosário M., Birchmeier W. (2006). Balancing cell adhesion and Wnt signaling, the key role of *β*-catenin. *Current Opinion in Genetics & Development*.

[26] Lu Y., Zhu X., Liang G. X. (2012). Apelin-APJ induces ICAM-1, VCAM-1 and MCP-1 expression via NF-*κ*B/JNK signal pathway in human umbilical vein endothelial cells. *Amino Acids*.

[27] Chang L., Karin M. (2001). Mammalian MAP kinase signalling cascades. *Nature*.

[28] Voraberger G., Schäfer R., Stratowa C. (1991). Cloning of the human gene for intercellular adhesion molecule 1 and analysis of its 5'-regulatory region. Induction by cytokines and phorbol ester. *Journal of Immunology*.

[29] Bonan S., Albrengues J., Grasset E. (2017). Membrane-bound ICAM-1 contributes to the onset of proinvasive tumor stroma by controlling acto-myosin contractility in carcinoma-associated fibroblasts. *Oncotarget*.

[30] Kovary K., Bravo R. (1992). Existence of different Fos/Jun complexes during the G0-to-G1 transition and during exponential growth in mouse fibroblasts: differential role of Fos proteins. *Molecular and Cellular Biology*.

[31] Ishida M., Ueki M., Morishita J., Ueno M., Shiozawa S., Maekawa N. (2015). T-5224, a selective inhibitor of c-Fos/activator protein-1, improves survival by inhibiting serum high mobility group box-1 in lethal lipopolysaccharide-induced acute kidney injury model. *Journal of Intensive Care*.

[32] Benedicto A., Romayor I., Arteta B. (2017). Role of liver ICAM-1 in metastasis. *Oncology Letters*.

[33] de Giorgio-Miller A., Bottoms S., Laurent G., Carmeliet P., Herrick S. (2005). Fibrin-induced skin fibrosis in mice deficient in tissue plasminogen activator. *The American Journal of Pathology*.

[34] Baroni G., Schuinski A., de Moraes T. P., Meyer F., Pecoits-Filho R. (2012). Inflammation and the peritoneal membrane: causes and impact on structure and function during peritoneal dialysis. *Mediators of Inflammation*.

[35] Ellis H., Harrison W., Hugh T. (1965). The healing of peritoneum under normal and pathological conditions. *The British Journal of Surgery*.

[36] Deng Z. H., Yin J. J., Luo W. (2018). The effect of earthworm extract on promoting skin wound healing. *Bioscience Reports*.

[37] Zhang M., Li X., Liu Y., Ye F., Qiu G. (2006). Effects of extract of dilong (pheretima) on the scalded skin in rats. *Journal of Traditional Chinese Medicine*.

[38] Liu C., Chen X., Pan Y., Liang H., Song S., Ji A. (2017). Antitumor studies of earthworm fibrinolytic enzyme component a from *Eisenia foetida* on breast cancer cell line MCF-7. *Indian Journal of Pharmaceutical Sciences*.

[39] Chang Y. M., Shih Y. T., Chen Y. S. (2011). Schwann cell migration induced by earthworm extract via activation of PAs and MMP2/9 mediated through ERK1/2 and p38. *Evidence-based Complementary and Alternative Medicine*.

[40] Fu Y. T., Sheu S. Y., Chen Y. S., Chen K. Y., Yao C. H. (2015). Porous gelatin/tricalcium phosphate/genipin composites containing lumbrokinase for bone repair. *Bone*.

[41] Roebuck K. A., Finnegan A. (1999). Regulation of intercellular adhesion molecule-1 (CD54) gene expression. *Journal of Leukocyte Biology*.

[42] Dalal P. J., Sumagin R. (2020). Emerging functions of ICAM-1 in macrophage efferocytosis and wound healing. *Journal of Cellular Immunology*.

[43] Meier H., Dietl K. H., Willital G. H. (1985). Initial clinical results of the prevention of intraoperative adhesions in children. *Langenbecks Archiv für Chirurgie*.

[44] Rivkind A. I., Lieberman N., Durst A. L. (1985). Urokinase does not prevent abdominal adhesion formation in rats. *European Surgical Research*.

