# Supplementary material for: Lumbrokinase, a Fibrinolytic Enzyme, Prevents Intra-Abdominal Adhesion by Inhibiting the Migrative and Adhesive Activities of Fibroblast via Attenuation of the AP-1/ICAM-1 Signaling Pathway
Source: Biomed Res Int. 2023 Jan 12;2023:4050730. doi: 10.1155/2023/4050730 (PMC9851794; doi:10.1155/2023/4050730)
Supplement: Supplementary Materials — Supplementary Material and Methods. Fibrin Plate Assay. Fibrinogen (#F3879) and thrombin (#T6884-250UN) were purchased from Sigma-Aldrich (St. Louis, MO, USA). Fibrinogen was diluted in phosphate-buffered saline (Welgene, Gyeongsan, Korea) to make 0.3% concentrated fibrinogen, which was incubated at 30°C for 60 min. After completely dissolving the fibrinogen, the fibrinogen solution was filtered through a 0.2 μm filter, and 10 μL of 20 U/mL thrombin was added to 15 mL of the fibrinogen solution. Next, 13 mL of 1% agarose solution was added to 15 mL of the fibrinogen-thrombin mixture [1, 2]. Afterward, 10 mL of the solution was clotted in a 100π Petri dish. The plates were allowed to stand at room temperature (25°C) for at least 30 min before being used. After clotting, holes were made using a Pasteur pipette. Next, 50 μL of the indicated concentration of lumbrokinase was dispensed into the holes of the plates, which were then incubated for the indicated hours at 37°C. Plasmin (P1867-500UG, Sigma, St. Louis, MO, USA) was used (0.1 U/mL) as a positive control. The fibrinolytic activity was calculated as the lytic area surrounding each well of the fibrin plate. The diameter of the area was measured with a ruler. Bleeding Time and Hematological Test. Bleeding time was measured using primary saline [3]. This test is performed by immersing the tip of the ear in 0.9% saline at 37°C for 2 min. Then, the tip is transfixed with a scalpel blade (approximately 0.5 cm), after which the cut ear tip is resubmerged in the warm saline, and the time to cessation of bleeding is recorded. A normal value is defined as <5 min, and an abnormal value is defined as >15 min. After measuring the bleeding time, whole blood was collected from the carotid artery. The tests of complete blood count and prothrombin time were performed in the laboratory of Chung-Ang University Hospital. Blood was collected into tubes containing ethylenediaminetetraacetic acid (EDTA) and tubes containing sodium citra [file 4050730.f1.zip › Supplementary data and Table -QCU.docx]

**Supplementary data**

**Supplementary Material and Methods**

*Fibrin plate assay*

Fibrinogen (#F3879) and thrombin (#T6884-250UN) were purchased from Sigma-Aldrich (St Louis, MO, USA). Fibrinogen was diluted in phosphate-buffered saline (Welgene, Gyeongsan, Korea) to make 0.3% concentrated fibrinogen, which was incubated at 30 ℃ for 60 min. After completely dissolving the fibrinogen, the fibrinogen solution was filtered through a 0.2-μm filter, and 10 μL of 20 U/mL thrombin was added to 15 mL of the fibrinogen solution. Next, 13 mL of 1% agarose solution was added to 15 mL of the fibrinogen-thrombin mixture.^1,2^ Afterward, 10 mL of the solution was clotted in a 100π Petri dish. The plates were allowed to stand at room temperature (25 °C) for at least 30 min before being used. After clotting, holes were made using a Pasteur pipette. Next, 50 μL of the indicated concentration of lumbrokinase was dispensed into the holes of the plates, which were then incubated for the indicated hours at 37 °C. Plasmin (P1867-500UG, Sigma, St. Louis, MO, USA) was used (0.1 U/mL) as a positive control. The fibrinolytic activity was calculated as the lytic area surrounding each well of the fibrin plate. The diameter of the area was measured with a ruler.

*Bleeding time and hematological test*

Bleeding time was measured using primary saline.^3^ This test is performed by immersing the tip of the ear in 0.9% saline at 37 °C for 2 min. Then, the tip is transfixed with a scalpel blade (approximately 0.5 cm), after which the cut ear tip is resubmerged in the warm saline, and the time to cessation of bleeding is recorded. A normal value is defined as <5 min, and an abnormal value is defined as >15 min. After measuring the bleeding time, whole blood was collected from the carotid artery. The tests of complete blood count and prothrombin time were performed in the laboratory of Chung-Ang University Hospital. Blood was collected into tubes containing ethylenediaminetetraacetic acid (EDTA) and tubes containing sodium citrate. The normal ranges of hematological parameters of male Sprague-Dawley rats are reported in the literature.^4^

**Supplementary Figure Legends**

**Figure S1.** Histological analysis of adhesion area between cecum and peritoneum. Two subjects were used in each group. The adhesion areas at one, two, and three weeks after tissue injuries were shown with hematoxylin and eosin (HE) staining (left panels) and Masson’s trichrome staining (right panels). Fibroblasts and inflammatory cells (arrow) were accumulated in the adhesion area in the 1-week tissues, and these were resolved in the two- and three-week tissues. This finding indicated that two weeks is the optimal time to observe tissue adhesion.

**Figure S2.** Gross scoring of adhesion two weeks after tissue injuries; the degree of intra-abdominal adhesion was measured by scoring the severity and area of adhesion. The severity of adhesion was as follows: 0, no adhesions; 1, filmy adhesions that are easily separated with blunt dissection or traction; 2, dense adhesions that are separated by blunt dissection but partly sharp dissection; 3, dense vascularized adhesions that are separated by sharp dissection only. The adhesion area of the initially injured area was assessed as follows: 0, no adhesion; 1, <25% of the traumatized area; 2, 25–50% of the traumatized area; 3, >50% of the traumatized area.

**Figure S3.** Fibrinolytic activity of lumbrokinase. (**A**) To validate the effect of lumbrokinase, the represented concentration of lumbrokinase was applied to the holes of the fibrin plate and incubated at 37 °C for the indicated time. The circles around the hole of the fibrin plate indicated fibrinolysis, and the lysis effect was assessed by measuring the diameter of the circles. Plasmin was used as a positive control. The result showed that lumbrokinase lyses fibrin in a dose-dependent manner. (**B**) To obtain the minimum concentration to lyse fibrin, low concentrations of lumbrokinase were applied. Treatment with 2,000 U/mL of lumbrokinase had no effect and 3,750 U/mL had minor effects. Therefore, 5,000 U/mL was used as the minimum concentration. Hyaluronate (HA) had no effect on fibrin lysis. (**C**) To assess whether hyaluronate influenced the lumbrokinase effect, cotreatment of lumbrokinase and hyaluronate was performed. The results showed that HA had no effect and did not compromise the lumbrokinase effect.

**Figure S4.** Coagulation profiles after lumbrokinase treatment. Bleeding time (**A**) and prothrombin time (**B**) (HA, hyaluronate).

**Supplementary Table**

**Table S1.** Laboratory results on day 7.

| Sample number | WBC  (10^3^/µL) | NEUT  (%) | LYMPH  (%) | MONO  (%) | EO  (%) | BASO  (%) | RBC  (10^6^/µL) | HGB  (g/dL) | HCT  (%) | MCV  (fL) | MCH  (pg) | MCHC  (g/dL) | PLT  (10^3^/µL) |
| --- | --- | --- | --- | --- | --- | --- | --- | --- | --- | --- | --- | --- | --- |
| Control-1 | 5.48 | 33.1 | 54.2 | 10.2 | 2 | 0.5 | 7.01 | 14.5 | 47.8 | 68.2 | 20.7 | 30.3 | 1329 |
| Control-2 | 4.85 | 30.2 | 51.8 | 12.4 | 5.4 | 0.2 | 5.88 | 11.9 | 39.1 | 66.5 | 20.2 | 30.4 | 1229 |
| 10,000 U/mL-1 | 9.06 | 22.8 | 65.5 | 6 | 5.6 | 0.1 | 7.24 | 15.1 | 47.2 | 65.2 | 20.9 | 32.0 | 465 |
| 10,000 U/mL-2 | 9.49 | 15.1 | 82.9 | 1.2 | 0.7 | 0.1 | 7.95 | 14.9 | 44.4 | 55.8 | 18.7 | 33.6 | 921 |
| 20,000 U/mL-1 | 9.32 | 19.4 | 77.4 | 1.3 | 1.8 | 0.1 | 8.19 | 15.4 | 46.3 | 56.5 | 18.8 | 33.3 | 994 |
| 20,000 U/mL-2 | 7.48 | 27.2 | 57.2 | 8.2 | 7.4 | 0 | 6.82 | 13.7 | 44.2 | 64.8 | 20.1 | 31.0 | 1261 |
| HA-1 | 8.23 | 42 | 49 | 6.6 | 2.3 | 0.1 | 6.26 | 13.2 | 42.2 | 67.4 | 21.1 | 31.3 | 666 |
| HA-2 | 4.78 | 35.4 | 51.5 | 12.3 | 0.4 | 0.4 | 6.4 | 13.2 | 43.7 | 68.3 | 20.6 | 30.2 | 459 |
| 5,000 U/mL+HA-1 | 4.87 | 13.1 | 82.8 | 2.7 | 1.4 | 0 | 7.1 | 14.8 | 48 | 67.6 | 20.8 | 30.8 | 1552 |
| 5,000 U/mL+HA-2 | 4.52 | 18.6 | 76.5 | 3.8 | 1.1 | 0 | 7.02 | 14.3 | 47.1 | 67.1 | 20.4 | 30.4 | 1062 |
| 10,000 U/mL+HA-1 | 14.68 | 19.8 | 77.7 | 1.2 | 1.2 | 0.1 | 7.37 | 14.6 | 43.7 | 59.3 | 19.8 | 33.4 | 1073 |
| 10,000 U/mL+HA-2 | 11.12 | 12.4 | 83.9 | 1.5 | 2.1 | 0.1 | 7.64 | 14 | 42.3 | 55.4 | 18.3 | 33.1 | 653 |
| 20,000 U/mL+HA-1 | 9.99 | 33.1 | 54.6 | 7.6 | 4.5 | 0.2 | 7.25 | 14.5 | 46 | 63.4 | 20 | 31.5 | 644 |
| 20,000 U/mL+HA-2 | 8.93 | 12.7 | 83.4 | 1.3 | 2.5 | 0.1 | 7.57 | 14.3 | 42.4 | 56 | 18.9 | 33.7 | 1221 |

WBC, white blood cells; NEUT, neutrophils; LYMPH, lymphocytes; MONO, monocytes; EO, eosinophils; BASO, basophils; RBC, red blood cells; HGB, hemoglobin; HCT, hematocrit; MCV, mean corpuscular volume; MCH, mean cell hemoglobin; MCHC, mean corpuscular hemoglobin concentration; PLT, platelets.

**References**

1. Astrup T, Müllertz S. The fibrin plate method for estimating fibrinolytic activity. *Arch Biochem Biophys*. 1952;40:346-351. https://doi.org/10.1016/0003-9861(52)90121-5

2. Ningthoujam DS, Thokchom S. Screening of fibrinolytic enzymes from microorganisms especially Actinomycetes from different biotopes in manipur. *Arch Clin Microbiol*. 2016;7:21. <https://doi.org/>10.4172/1989-8436.100050

3. Scola MR, Baggesen LM, Nichols TC, Key NS, Gallippi CM. A review of current methods for assessing hemostasis in vivo and introduction to a potential alternative approach. *Thromb Res*. 2012;129:S57-S61. https://doi.org/10.1016/j.thromres.2012.02.035

4. He Q, Su G, Liu K, Zhang F, Jiang Y, Gao J, et al. Sex-specific reference intervals of hematologic and biochemical analytes in Sprague-Dawley rats using the nonparametric rank percentile method. *PLoS One*. 2017;12:e0189837. https://doi.org/10.1371/journal.pone.0189837
